# Intermittent versus continuous energy restriction on weight loss and cardiometabolic outcomes: a systematic review and meta-analysis of randomized controlled trials

**DOI:** 10.1186/s12967-018-1748-4

**Published:** 2018-12-24

**Authors:** Iolanda Cioffi, Andrea Evangelista, Valentina Ponzo, Giovannino Ciccone, Laura Soldati, Lidia Santarpia, Franco Contaldo, Fabrizio Pasanisi, Ezio Ghigo, Simona Bo

**Affiliations:** 1 Interuniversity Center for Obesity and Eating Disorders, Department of Medicine and Surgery, Federico II University Hospital, Pansini, 5, Naples, 80131 Italy; 2 Unit of Clinical Epidemiology, CPO, “Città della Salute e della Scienza” Hospital of Turin, Turin, Italy; 3 Department of Medical Sciences, University of Turin, c.so AM Dogliotti 14, 10126 Turin, Italy; 4 Department of Health Sciences, University of Milan, Milan, Italy

**Keywords:** Continuous energy restriction, Intermittent energy restriction, Fasting glucose, Triglycerides, Weight loss

## Abstract

**Background:**

This systematic review and meta-analysis summarized the most recent evidence on the efficacy of intermittent energy restriction (IER) versus continuous energy restriction on weight-loss, body composition, blood pressure and other cardiometabolic risk factors.

**Methods:**

Randomized controlled trials were systematically searched from MEDLINE, Cochrane Library, TRIP databases, EMBASE and CINAHL until May 2018. Effect sizes were expressed as weighted mean difference (WMD) and 95% confidence intervals (CI).

**Results:**

Eleven trials were included (duration range 8–24 weeks). All selected intermittent regimens provided ≤ 25% of daily energy needs on “fast” days but differed for type of regimen (5:2 or other regimens) and/or dietary instructions given on the “feed” days (ad libitum energy versus balanced energy consumption). The intermittent approach determined a comparable weight-loss (WMD: − 0.61 kg; 95% CI − 1.70 to 0.47; p = 0.87) or percent weight loss (WMD: − 0.38%, − 1.16 to 0.40; p = 0.34) when compared to the continuous approach. A slight reduction in fasting insulin concentrations was evident with IER regimens (WMD = − 0.89 µU/mL; − 1.56 to − 0.22; p = 0.009), but the clinical relevance of this result is uncertain. No between-arms differences in the other variables were found.

**Conclusions:**

Both intermittent and continuous energy restriction achieved a comparable effect in promoting weight-loss and metabolic improvements. Long-term trials are needed to draw definitive conclusions.

**Electronic supplementary material:**

The online version of this article (10.1186/s12967-018-1748-4) contains supplementary material, which is available to authorized users.

## Background

In the last decade, much interest has been focused on dietary strategies that manipulate energy intake unconventionally, known as intermittent fasting or intermittent energy restriction (IER) [1–4]. This dietary approach has gained greater attention and popularity as a way for losing weight alternative to the conventional weight-loss diets, characterized by continuous (non-intermittent) energy restriction (CER). The two most popular forms of IER are: the 5:2 diet characterized by two consecutive or non-consecutive “fast” days and the alternate-day energy restriction, commonly called alternate-day fasting, alternate-day modified fasting, or every-other-day fasting, consisting of a ‘‘fast” day alternated with a ‘‘feed” day [5]. Commonly, during “fast” days, the energy intake is severely restricted, ranging from complete abstinence from foods to a daily maximum intake roughly corresponding to 75% energy restriction. Therefore, the term “fast” often does not involve a true complete abstinence from caloric intake. The term IER will be used to describe all intermittent energy-restricted/fasting regimens.

The time-restricted feeding [2, 6–9] and the very-low-calorie or energy diets [2, 3] are other types of dietary interventions which were often included in previous systematic reviews and meta-analyses on IER. Indeed, in the former, individuals are allowed to eat within a specific range of time, thus, every day there is a period without food intake, varying from 12 to 21 h [10–12] (i.e. the Muslim Ramadan). On the other hand, there is no daily intermittency in a very-low-calorie-diet, although the overall energy intake may be similar to those of the IER regimens [13].

To the best of our knowledge, an overall evaluation of the impact of IER on multiple metabolic variables, on percent body fat changes, and on the effects of balanced versus ad libitum “feed” days, as well as on the benefits of the different “fasting” regimens is at present lacking.

The primary objective of this systematic review and meta-analysis was to update the efficacy of IER on weight loss, limiting the analyses to regimens which actually included a weekly intermittent energy restriction, i.e. from 1 up to 6 “fast days” per week. Furthermore, the impact of IER on fat mass (FM), fat free mass (FFM), arterial blood pressure (BP) and other cardiometabolic risk factors was assessed. The effects of IER according to the specific type of nutritional regimen on all these outcomes were evaluated too.

## Materials and methods

We followed the Preferred Reporting Items for Systematic Reviews and Meta-Analyses (PRISMA) guidelines in the reporting of this study [14].

### Search strategy

The following electronic databases were queried using a combination of search terms until the 31th of May 2018: PubMed (National Library of Medicine), the TRIP database, the Cochrane Library, EMBASE, and Cumulative Index to Nursing and Allied Health Literature (CINAHL). The construction of the search strategy was performed using database specific subject headings and keywords. Both medical subject headings (MeSH) and free text search terms were employed. Restrictions to human studies were placed.

The search terms included combinations of “intermittent fasting” or “alternate day fasting” or “intermittent energy restriction” or “periodic fasting”, and weight loss, weight gain, obesity, weight, fat mass, blood pressure, blood glucose, insulin, insulin-resistance, insulin sensitivity, glycated hemoglobin A1c (HbA1c), type 2 diabetes mellitus (T2DM), cholesterol, and triglycerides (free-term and MESH as possible) (Additional file 1). These search strategies were implemented by hand searching the references of all the included studies and systematic reviews on the field.

### Study selection

We included studies with the following characteristics: (1) randomized controlled trials (RCTs); (2) a detailed description of the IER regimen; (3) 75% of energy restriction on “fast” days, with a maximum cut-off of 500/660 kcal/day for females/males, respectively; (4) weekly intermittency of energy restriction (from 1 up to 6 “fast” days per week); (5) trial duration > 4 weeks; (6) containing as comparator a group on a CER regimen and (7) including changes in body weight or percent body weight as one of the study’s outcome.

We excluded studies with the following characteristics: (i) uncontrolled trials or study design other than RCTs; (ii) studies not including body weight as an outcome and/or lacking sufficient information on weight change; (iii) including time restricted feeding intervention; (iv) reporting very-low-calorie or fasting regimens for > 6 days consecutive/week; and (v) providing > 500–660 kcal/day or not reporting the amount of calorie prescribed on “fast” days.

In trials with multiple interventional arms (i.e. exercise arm, intervention arm with specific diets), the IER and the CER arms were considered, while other arms were not analyzed, since out of the scope of this review.

Two authors (IC, SB) separately screened abstracts for their inclusion or exclusion; retrieving full text articles from potentially relevant abstracts. Any discrepancy about inclusion was resolved by discussing with a third author (AE).

### Outcomes

The primary outcome of the review was evaluating changes in body weight or in percent body weight. Secondary outcomes were: changes in body mass index (BMI), waist circumference, FM, FFM, arterial BP, and the blood values of fasting glucose and insulin, insulin resistance, insulin sensitivity, HbA1c, total cholesterol, HDL- and LDL-cholesterol, and triglycerides. The changes of these outcomes according to the specific type of IER regimen were also evaluated.

### Data collection and extraction

From each included study, the following information were extracted (1) first author name and year of publication; (2) study design; (3) inclusion criteria of participants; (4) trial duration; (5) number of subjects enrolled in each arm; (6) type of dietary intervention; (7) age, gender, BMI of participants; (8) body composition (FM and FFM); (9) systolic (SBP) and diastolic blood pressure (DBP); (10) blood concentrations of fasting glucose, HbA1c, insulin, total cholesterol, HDL-cholesterol, LDL-cholesterol, and triglycerides; (11) Homeostasis Model Assessment-Insulin Resistance (HOMA-IR) and insulin-sensitivity index (Si).

### Risk of bias assessment

All studies were independently assessed by two authors (IC, SB) using the “Risk of bias” tool developed by the Cochrane Collaboration for RCTs [15]. The items used for the assessment of each study were the following: adequacy of sequence generation, allocation concealment, blinding, addressing of dropouts (incomplete outcome data), selective outcome reporting, and other potential sources of bias. A judgment of “L” indicated low risk of bias, “H” indicated high risk of bias, and “unclear” indicated an unclear/unknown risk of bias. The possible disagreements were resolved by consensus, or with consultation with a third author (AE).

### Data synthesis

Data synthesis was performed only for the outcomes which were reported by > 3 trials.

The pooled effect sizes were expressed as weighted mean differences (WMD) and 95% confidence interval (CI) between IER and CER arms of the mean outcome values measured at the end of follow-up.

The mean difference of changes from baseline was estimated for each study on the basis of reported baseline and follow-up measurements. If the standard deviation for change from baseline was not reported, we imputed missing values assuming a within-patient correlation from baseline to follow-up measurements of 0.8 as suggested in the Cochrane handbook [16]. When between-arms mean differences on change from baseline were already estimated [17], those data were included. For the relative weight change from baseline, the non-reported standard deviations were imputed using the mean standard deviation of the available studies.

Random-effects models were applied to provide a summary estimate.

Inter-study heterogeneity was assessed using Cochrane Q statistic and quantified by I^2^ test [18].

Subgroup analyses for all outcomes were performed based on the different dietary regimen of the “feed” days (balanced vs. ad libitum food intake) and the effects of the different regimens of “fasting” (5:2 vs. the other regimens). Weighting of studies was done using generic inverse variance method.

In order to evaluate the influence of each study on the overall effect size, sensitivity analysis was conducted using the one-study remove (leave-one-out) approach.

Potential publication bias was explored using visual inspection funnel plot asymmetry and Egger’s weighted regression tests.

Meta-analyses were performed by using the Stata Metan package (Stata Statistical Software, Release 13; StataCorp LP, College Station, TX); meta-regressions and Egger’s weighted regression tests for publication bias were performed using the metafor package (version 1.9-7) for R (version 3.1.2, R Foundation for Statistical Computing, Vienna, Austria).

## Results

### Included studies

**Fig. 1:**
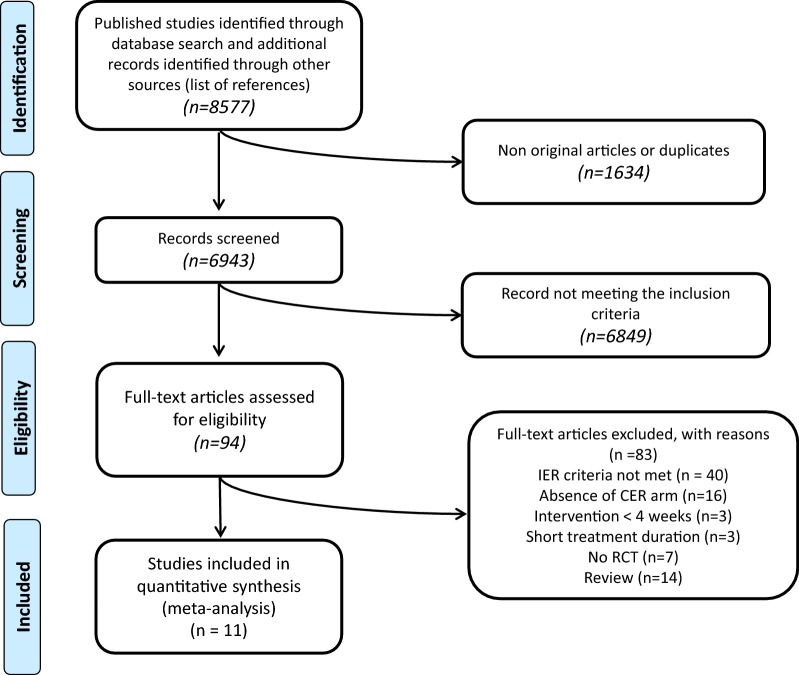
Flow of the study

The initial literature search identified 8577 records. After removing duplicates, 6943 records were screened, and, after excluding articles not meeting the inclusion criteria, 94 records were assessed for eligibility. After further analysis and quality assessment, a total of 11 studies were selected for the systematic review and meta-analysis (Fig. 1). All studies identified were RCTs, reporting an IER arm and a CER arm comparison; the corresponding details are shown in Table 1. Data relative to participants involved in exercise-only arms [19] or in high-protein dietary intervention [20] were not considered, because not pertinent to the aims of the study.

**Table 1:** Characteristics of the included studies

Author (year) [ref]	Study design	Participants	Trial duration	N	Study groups	Age (years)	Males (n)	BMI (kg/m^2^) Waist-c (cm)	FM FFM (kg)	SBP DBP (mmHg)	Fasting glucose (mg/dL) Hb1Ac (%)	Fasting insulin (µU/mL) HOMA-IR (mmol/L * µU/mL)	Total chol (mg/dL)	HDL-c (mg/dL)	LDL-c (mg/dL)	TG (mg/dL)
Antoni (2018) [22]	RCT 2 parallel arms	BMI > 25 kg/m^2^ Age 18–65 years No comorbidity	8–10 weeks	15^a^	IER (2 days/week) = 25% of the energy need on 2 consecutive fast days and energy according to needs on feed days	42 ± 4	7	30 ± 1	31 ± 2	123 ± 3	79 ± 2	11 ± 1	162 ± 12	42 ± 4	100 ± 12	97 ± 9
								102 ± 3	58 ± 3	74 ± 3	ND	1.6 ± 0.2				
				12^a^	CER: 600 kcal less than estimated needs	48 ± 3	6	31 ± 1	34 ± 3	115 ± 3	79 ± 4	9 ± 1	162 ± 12	39 ± 4	104 ± 8	80 ± 9
								102 ± 2	56 ± 3	75 ± 3	ND	1.3 ± 0.1				
Carter (2016) [24]	RCT 2 parallel arms	T2DM BMI ≥ 27 kg/m^2^ Age ≥ 18 years No comorbidity	12 weeks	31	IER (2 days/week) = 400–600 kcal/day on fast days and no restriction on feed days	61 ± 8	16	35 ± 5	38 ± 9	134 ± 17	ND	ND	ND	ND	ND	ND
								ND	55 ± 11	84 ± 10	7 ± 1					
				32	CER = 1200–1500 kcal/day every day	62 ± 9	14	36 ± 5	40 ± 11	138 ± 15	ND	ND	ND	ND	ND	ND
								ND	54 ± 9	90 ± 11	8 ± 1					
Catenacci (2016) [27]	RCT 2 parallel arms	BMI ≥ 30 kg/m^2^ Age 18–55 years Non-smokers No diabetes, CV diseases, major comorbidity	8 weeks	15	IER (3 days/week) = 0 kcal/day on fast days alternate and energy according to needs, but with the chance to ask for more food, on feed days	40 ± 10	3	36 ± 4	38 ± 8	ND	88 ± 7	13 ± 6	170 ± 33	38 ± 8	100 ± 31	143 ± 56
								ND	53 ± 9		ND	ND				
				14	CER = 400 kcal less than needs	43 ± 8	3	40 ± 6	49 ± 10	ND	92 ± 8	19 ± 6	171 ± 36	39 ± 7	104 ± 29	140 ± 43
								ND	61 ± 12		ND	ND				
Conley (2018) [23]	RCT 2 parallel arms	Men with BMI ≥ 30 kg/m^2^ Age 55–75 years No diabetes No high alcohol intake or major comorbidity	24 weeks	12	IER (2 days/week) = 600 kcal/day on 2 non-consecutive fast days and energy ad libitum on feed days	68 ± 3	12	33 ± 2	ND	142 ± 14	108 ± 27	ND	151 ± 35	45 ± 12	77 ± 31	168 ± 53
								114 ± 5		84 ± 10	ND					
				12	CER = 500 kcal less than needs	67 ± 4	12	36 ± 4	ND	150 ± 18	110 ± 31	ND	166 ± 39	46 ± 12	98 ± 35	212 ± 150
								123 ± 10		88 ± 14	ND					
Coutinho (2017) [28]	RCT 2 parallel arms	BMI = 30–40 kg/m^2^ Age 18–65 years Inactive, no menopause or major comorbidity	12 weeks	18	IER (3 days/week) = 550–660 kcal/day on fast days and energy according to needs on feed days	39 ± 11	4	36 ± 3	47 ± 8	ND	ND	ND	ND	ND	ND	ND
								ND	60 ± 12							
				17	CER = 33% of energy restriction every day	39 ± 9	2	35 ± 4	43 ± 8	ND	ND	ND	ND	ND	ND	ND
								ND	55 ± 9							
Harvie (2011) [26]	RCT 2 parallel arms	BMI = 24–40 kg/m^2^ Premenopausal women Age 30–45 years Non-smokers, not using OC, no major comorbidity	24 weeks	53	IER (2 days/week) = 25% of the energy need on 2 consecutive fast days and energy according to needs on feed days	40 ± 4	0	31 ± 5	34 (31–36)	115 (111–119)	87 (85–88)	7 (6–8)	197 (189–197)	58 (54–58)	119 (112–127)	106 (88–124)
								102 (98–105)	48 (46–49)	77 (74–79)	ND	1.5 (1.3–1.8)				
				54	CER = 25% energy restriction every day	40 ± 4	0	31 ± 5	35 (32–39)	117 (113–120)	87 (83–88)	7 (6–9)	200 (193–208)	62 (54–66)	119 (108–127)	115 (97–124)
								102 (99–106)	49 (48–50)	75 (72–78)	ND	1.6 (1.3–1.8)				
Harvie (2013) [20]	RCT 3 parallel arms	Women with BMI = 24–45 kg/m^2^ or body fat > 30% of BW Age 30–45 years No major comorbidity	12 weeks	37	IER (2 days/week) = 25% of the energy need on 2 consecutive fast days and energy according to needs on feed days	46 ± 8	0	30 ± 4	31 (28–34)	115 (111–125)	88 (85–90)	6 (5–8)	204 (191–229)	50 (49–59)	128 (116–139)	088 (75–102)
								101 (97–104)	48.5 (46–50)	ND	5	1.6 (1.3–1.9)				
				38	IER as above plus unlimited proteins and fats (non-SFA) on fast days	49 ± 7	0	31 ± 6	34 (30–37)	130 (115–138)	90 (86–92)	7 (6–9)	221 (206–237)	55 (51–59)	144 (130–159)	95 (81–109)
								104 (99–109)	49 (47–51)	ND	6	1.9 (1.5–2.2)				
				40	CER = daily 25% restriction every day	48 ± 8	0	32 ± 6	36 (32–39)	124 (116–131)	90 (86–92)	7 (6–9)	205 (193–218)	51 (48–55)	129 (118–139)	97 (83–111)
								106 (102–110)	50 (48–52)	ND	6	1.8 (1.5–2.2)				
Sundfor (2018) [21]	RCT 2 parallel arms	BMI = 30–45 kg/m^2^ Age 21–70 years Waist ≥ 84/90 cm (male/female) plus another component of the metabolic syndrome No comorbidity or alcohol/drug abuse	24 weeks	54	IER (2 days/week) = 400–600 kcal/day on 2 non-consecutive fast days and energy as usual on feed days	50 ± 10	28	35 ± 4	ND	129 ± 13	104 ± 22	ND	192 ± 35	47 ± 13	126 ± 32	162 ± 73
								116 ± 10		88 ± 8	6 ± 1					
				58	CER = 400–600 kcal less than needs	48 ± 12	28	35 ± 4	ND	128 ± 13	103 ± 13	ND	197 ± 34	45 ± 10	133 ± 32	137 ± 60
								116 ± 10		86 ± 9	6 ± 1					
Trepanowski (2017) [17]	RCT 3 parallel arms	BMI = 25.0–39.9 kg/m^2^ Age 18–65 years Non-smokers, inactive No menopause, diabetes, CV diseases	24 weeks	34	IER (alternate d/week) = 25% of the energy need on fast days and 125% of energy needs on feed days	44 ± 10	4	34 ± 4	38 ± 7	124 ± 12	90 ± 12	16 ± 14	188 ± 35	57 ± 14	111 ± 13	101 ± 59
								ND	55 ± 9	83 ± 9	ND	ND				
				35	CER = daily 25% restriction every day	43 ± 12	6	35 ± 4	40 ± 7	122 ± 17	92 ± 18	20 ± 18	184 ± 35	53 ± 11	112 ± 31	97 ± 27
								ND	58 ± 12	80 ± 11	ND	ND				
				31	C = no dietary intervention	44 ± 11	4	34 ± 4	36 ± 10	121 ± 16	87 ± 8	16 ± 9	190 ± 30	59 ± 13	112 ± 31	98 ± 43
								ND	53 ± 10	81 ± 11	ND	ND				
Varady (2011) [19]	RCT 4 parallel arms	BMI = 25–39.9 kg/m^2^ Age 35–65 years Non-smokers, inactive No diabetes, CV diseases	12 weeks	15	IER (3 days/week) = 25% of the energy need on fast days and ad libitum on feed days	47 ± 2	3	32 ± 2 ND	ND	ND	ND	ND	ND	51 ± 3	141 ± 9	ND
				15	CER = 25% energy restriction every day	47 ± 3	2	32 ± 2 ND	ND	ND	ND	ND	ND	60 ± 6	137 ± 9	ND
				15	Moderate exercise program, no diet intervention	46 ± 3	2	33 ± 1 ND	ND	ND	ND	ND	ND	51 ± 4	122 ± 9	ND
				15	C = no dietary/exercise intervention	46 ± 3	2	32 ± 2 ND	ND	ND	ND	ND	ND	57 ± 3	136 ± 10	ND
Williams (1998) [25]	RCT, 3 parallel Arms	T2DM Age 30–70 years BW > 20% ideal Not currently on insulin, no liver, renal, heart diseases	20 weeks	18	IER (1 day/week) = 400–600 kcal/day on fast day and 1500–1800 kcal/day on feed days	51 ± 8	9	35 ± 5	ND	ND	177 ± 56	20 ± 11	215 ± 37	41 ± 8	132 ± 35	197 ± 83
								ND			8 ± 2	ND				
				18	IER (5 days/week) = 400–600 kcal/day on fast day every 5 weeks and 1500–1800 kcal/day on feed days	50 ± 9	7	37 ± 5	ND	ND	182 ± 58	17 ± 7	208 ± 39	42 ± 7	131 ± 29	203 ± 239
								ND			8 ± 2	ND				
				18	CER = 1500–1800 kcal/day every day	54 ± 7	7	35 ± 5	ND	ND	184 ± 61	22 ± 9	218 ± 42	46 ± 11	127 ± 48	167 ± 89
								ND			8 ± 2	ND				

*BMI* body mass index, *BW* body weight, *Chol* cholesterol, *CV* cardiovascular, *CER* continuous energy restriction, *DBP* diastolic blood pressure, *FFM* fat free mass, *FM* fat mass, *IER* intermittent energy restriction, *HDL-c* high density lipoprotein-cholesterol, *LDL-c* low density lipoprotein-cholesterol, *ND* no data, *OC* oral contraceptives, *RCT* randomized controlled trial, *SFA* saturated fatty acids, *SBP* systolic blood pressure, *TG* triglycerides, T2DM type 2 diabetes mellitus, *Waist-c* waist circumference, *Wk* week

^a^Not available the baseline data of all the randomized patients; the study reported the baseline characteristics of the study completers only

### Characteristics of the studies

The total number of subjects included in the present analysis was 630 at enrolment. During the course of the trials, 102 patients dropped out. Drop-out rates ranged from about 2% [21] to 38% for IER arms [22] and from 0% [23] to 50% [22] for CER. The number of participants analyzed at the end of the RCTs was 528.

There was a greater number of women among participants, with the exception of 3 studies with a balanced number between men and women [21, 22, 24] and 1 enrolling only men [23]. Participants were individuals with overweight/obesity; in 2 RCTs patients with T2DM were selected [23, 25], and in 1 RCT patients with multiple dysmetabolic conditions were enrolled [21]. In all RCTs except for 2 [23, 25], participants with a stable weight before the beginning of the study, without history of bariatric surgery, and without drugs impacting on weight or the other study outcomes, were studied.

Trials were performed in UK [20, 22, 26], in USA [17, 19, 25, 27], in Australia [23, 24], and Norway [21, 28]. The duration of the studies ranged from 8 weeks [27] to 24 weeks [17, 21, 23, 26].

### Dietary intervention

Four studies prescribed alternating “fast” and “feed” days [17, 19, 27, 28]. Six studies used 2 “fast” days and 5 “feed” days per week (5:2 diet) [21–24, 26]. In 1 RCT, 5 consecutives “fast” days were prescribed before a 1 “fast” day/week regimen per 15 weeks in the IER arm, while the other arm (5 “fast” days every 5 weeks) was not considered, since no intermittence within the same week was present [25]. On “fast” days, diets provided a maximum of 660 kcal/day. In 2 studies, participants were instructed to consume their meals between 12:00 p.m. and 2:00 p.m. on “fast” days to ensure that subjects underwent the same duration of fasting [17, 19]. In 4 studies, meals of “fast” days were partially [17, 25] or totally supplied [19, 27]. In 1 study, a commercially available very-low energy formula-based food was assigned in the “fast” days [22].

On “feed” days, 6 studies prescribed healthy and balanced eating pattern, according to the energy requirements [17, 20, 22, 25, 26, 28], 4 allowed for ad libitum food intake based on the participants’ usual eating [19, 21–24] and 1 provided a diet based on the energy requirements but allowing the access to 5–7 optional food modules (200 kcal each) [27]. In the comparator arms, energy was restricted by approximately 25% of the daily energy requirements in all studies (CER arms).

### Dietary compliance and energy intake assessment

Six studies specifically assessed the compliance to the diet and the overall energy intake in both arms by filling 7-day food records at different time points [17, 20–22, 26, 28]. In 1 study, dieticians evaluated adherence by using patients’ self-recorded dietary diaries and diet histories taken during their dietetic appointments [23]. Either similar adherence between IER and CER [20, 21, 23, 26, 28], a lower [17] or a higher [22] adherence in the IER arms were reported. Adherence to the recommendations in the IER arms ranged from 64% [26] to 93% [22] at the end of the RCTs, but data were difficult to compare because of their incompleteness and the different methods employed to evaluate the compliance.

### Risk of bias assessment

Some of the analyzed trials were characterized by the lack of information about the randomization procedures (Additional file 2). If blinding of participants was not feasible owing to the nature of the interventions, data about blinding of the personnel performing the laboratory or statistical analyses were always unknown, except for 1 study [20]. Dropouts were higher in the IER arms [17, 26, 28] or in the CER arms [20, 22, 24, 25], thus introducing a possible selection bias between-arms, but intention-to treat analyses were performed by all studies, except for 1 RCT [22], where data of the completers only have been reported. Finally, most trials appeared to be free of selective outcome reporting and of other sources of bias, apart from 1, where body weight at baseline was not reported [19].

### Meta-analysis

All the outcomes of interest of this systematic review are reported in Additional file 3. Data synthesis was performed for the outcomes reported by > 3 trials, therefore data relative to Si values were not pooled.

### Weight loss

**Fig. 2:**
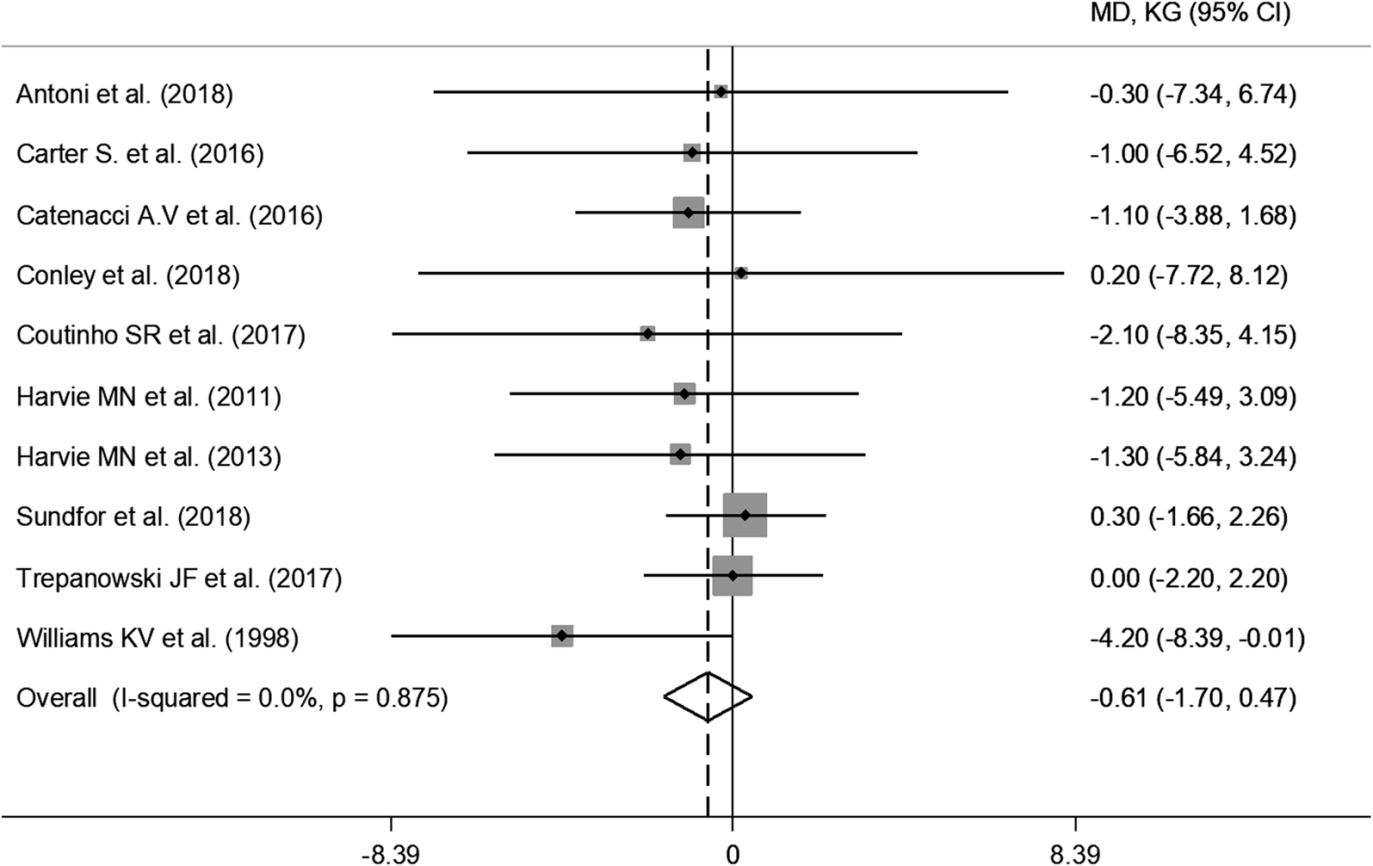
Meta-analysis of the effects of intermittent energy restriction versus continuous energy restriction on weight loss. MD (mean difference) indicates the mean difference on change from baseline of the IER vs. the CER arms. The plotted points are the mean differences and the horizontal error bars represent the 95% confidence intervals. The grey areas are proportional to the weight of each study in the random-effects meta-analysis. The vertical dashed line represents the pooled point estimate of the mean difference. The solid black line indicates the null hypothesis (MD = 0)

All RCTs reported weight loss in the IER arms during the intervention, ranging from 5.2% [19] of initial weight to 12.9% [28], while in the CER arms, changes ranged from 4.3% [20] to 12.1% [28] (Additional file 3). Pooled data from random-effect analysis did not show a significant effect of IER on weight loss (WMD: − 0.61 kg, 95% CI − 1.70 to 0.47; p = 0.27) (Fig. 2). The estimated effect on body weight did not change in the leave-one-out sensitivity analysis (data not shown).

Subgroup analyses based on the type of regimen (5:2 vs. other regimens) as well as on the dietary characteristics of the “feed” days of the IER interventions (ad libitum vs. balanced food intake) showed consistent results, as reported in Additional file 4. Analyses were repeated after the exclusion of the trial prescribing 5 consecutives “fast” days and then 1 “fast” day/week per 15 weeks [25], and the results did not change (WMD: − 0.36 kg, 95% CI − 1.48 to 0.77; p = 0.54). Finally, the RCT reporting the percent relative variations of the endpoints only [19] was included in the analyses, and the estimated effect size of weight change did not show any between-arms difference (WMD: − 0.08, 95% CI − 0.23 to 0.07; p = 0.29).

Similarly, the percent weight loss was similar in both arms (WMD: − 0.38%, 95% CI − 1.16 to 0.40; p = 0.34) and the results did not differ either in the subgroup analyses (Additional file 5) or in the leave-one-out sensitivity analysis.

### Other anthropometric measures

Seven out of the 11 included RCTs reported changes in FM and FFM [17, 20, 22, 24, 26–28]. FM was measured by different methods: body impedance analysis (BIA) [20, 22]; dual X-ray absorptiometry (DXA) [17, 24, 27]; impedance [26]; air displacement plethysmography [28]. Pooled results showed no difference between-arms in FM (WMD: − 0.23 kg, 95% CI − 1.23 to 0.77; p = 0.66) as well as in FFM (WMD: − 0.22 kg, 95% CI − 1.01 to 0.56; p = 0.58), as shown in Additional file 6. Those results were consistent both at subgroup analyses and at sensitivity analyses. Five RCTs assessed waist circumference [20–23, 26] without showing any differences between arms (WMD: − 0.17 cm; 95% CI − 1.74 to 1.39; p = 0.83).

### Cardiometabolic biomarkers

**Fig. 3:**
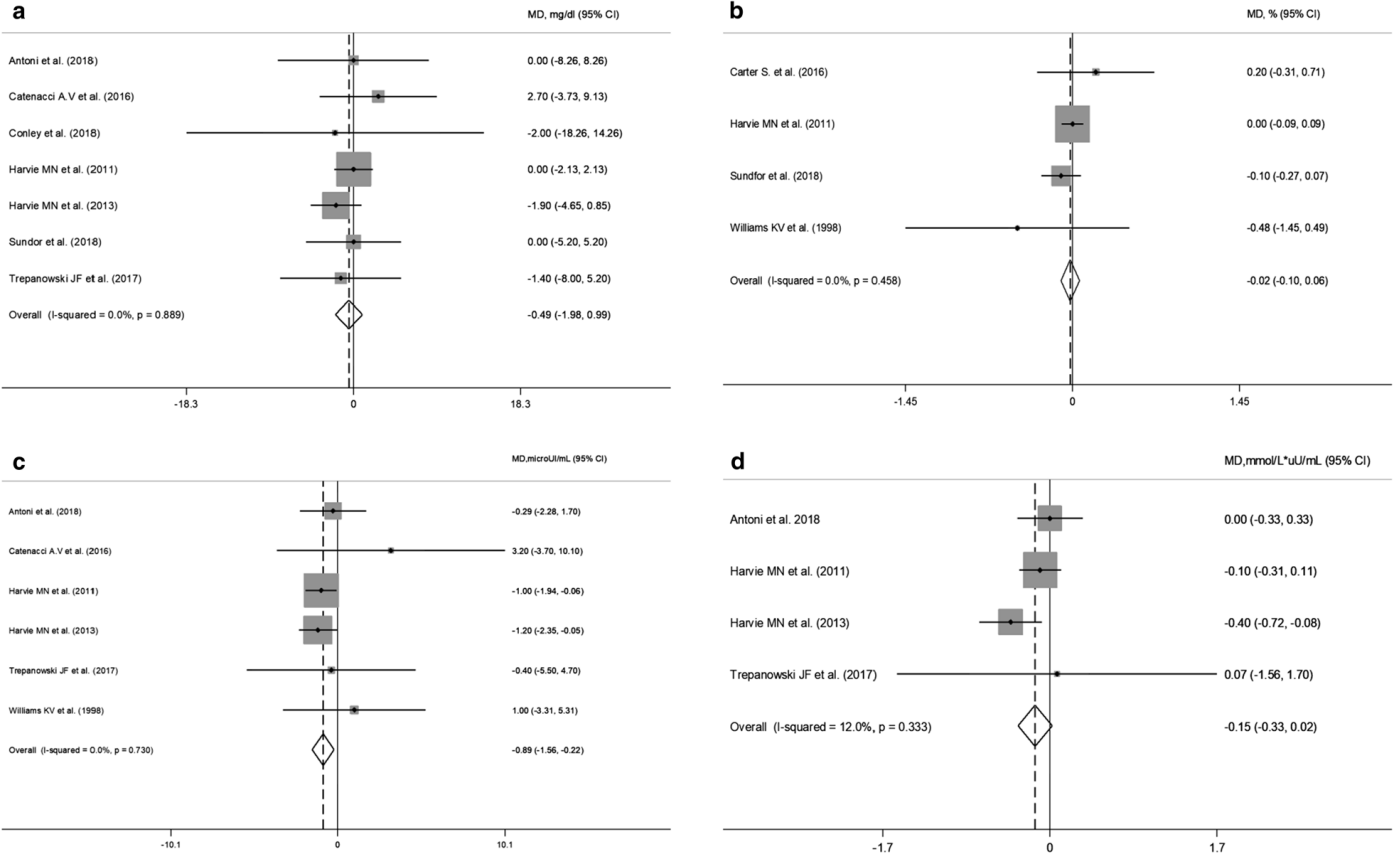
Meta-analysis of the effects of intermittent energy restriction versus continuous energy restriction on fasting glucose (**a**), HbA1c (**b**), insulin (**c**) and HOMA-IR (**d**) values. MD (mean difference) indicates the mean difference on change from baseline of the IER vs. the CER arms. The plotted points are the mean differences and the horizontal error bars represent the 95% confidence intervals. The grey areas are proportional to the weight of each study in the random-effects meta-analysis. The vertical dashed line represents the pooled point estimate of the mean difference. The solid black line indicates the null hypothesis (MD = 0)

Pooled data obtained from glucose, HbA1c, insulin and HOMA-IR are presented in Fig. 3a–d respectively. Changes in fasting glucose and HbA1c values were reported respectively in 7 [17, 20–23, 26, 27] and 4 [21, 24–26] trials. Random-effect analysis showed no difference either on glucose (WMD: − 0.49 mg/dL, 95% CI − 1.98 to 0.99; p = 0.51) or HbA1c (WMD: − 0.02%, 95% CI − 0.10 to 0.06; p = 0.62) changes in the IER when compared to CER arms with consistent results in subgroup/sensitivity analyses.

On the contrary, fasting insulin values were significantly reduced with IER (WMD = − 0.89 µU/mL; 95% CI − 1.56 to − 0.22; p = 0.009; I^2^ = 0%) and the estimated effect appeared robust in the leave-one-out sensitivity analysis (data not shown). Moreover, subgroup analyses showed that the 5:2 regimens were associated with increased insulin reductions (WMD: − 0.99 µU/mL; 95% CI − 1.67 to − 0.30; p = 0.005; I^2^ = 0) (Additional file 7). All the RCTs evaluating fasting insulin values included a balanced energy regimen for the “feed” days. HOMA-IR values were reduced, though not significantly, in the IER regimens (WMD = − 0.15 mmol/L × µU/mL; 95% CI − 0.33 to 0.02; p = 0.09).

Only 1 RCT evaluated insulin sensitivity (Si) by a frequently sampled intravenous glucose tolerance [21], without between-arms differences.

**Fig. 4:**
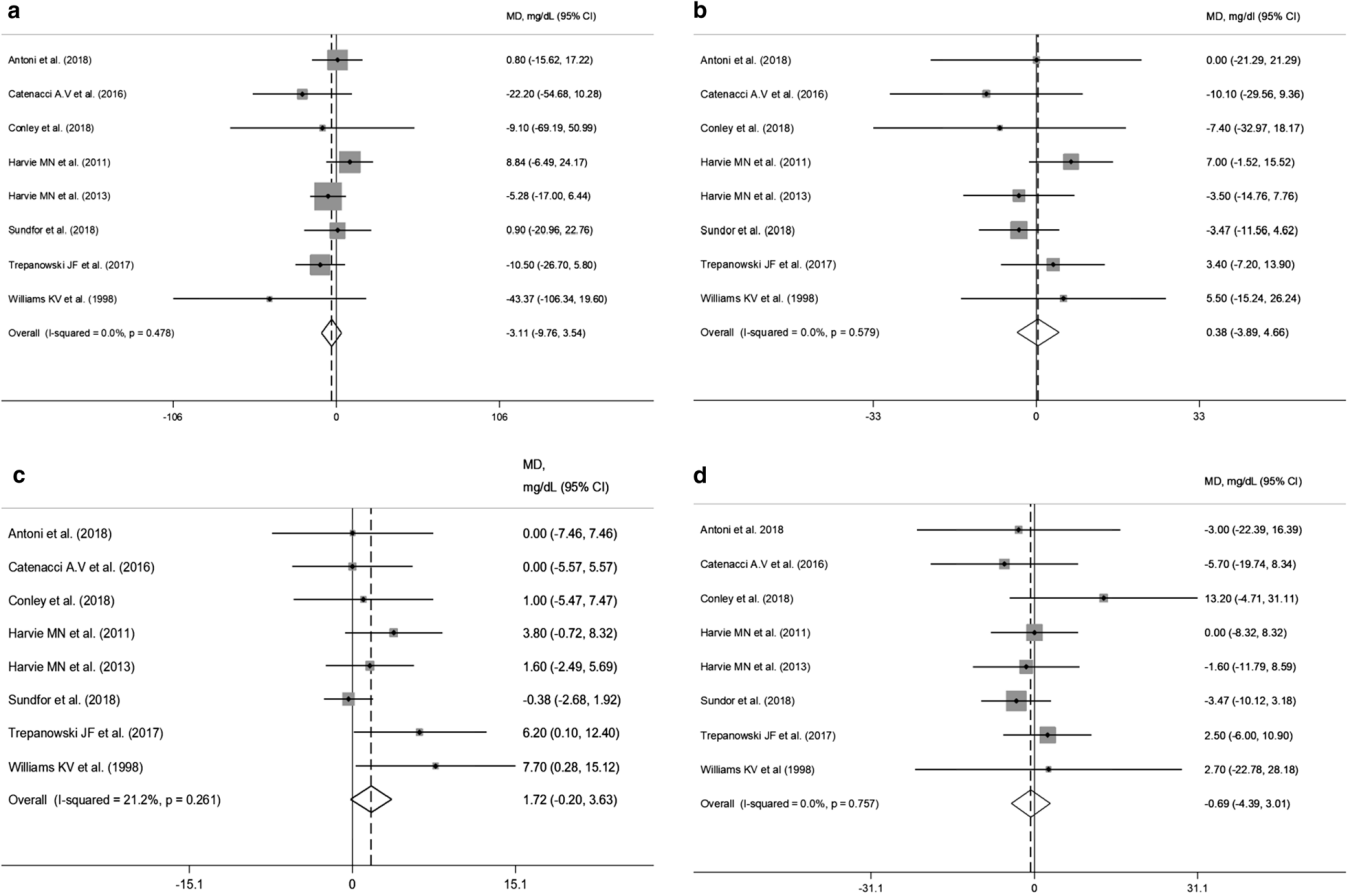
Meta-analysis of the effects of intermittent energy restriction versus continuous energy restriction on triglycerides (**a**), total cholesterol (**b**), HDL-cholesterol (**c**) and LDL-cholesterol (**d**) values. MD (mean difference) indicates the mean difference on change from baseline of the IER vs. the CER arms. The plotted points are the mean differences and the horizontal error bars represent the 95% confidence intervals. The grey areas are proportional to the weight of each study in the random-effects meta-analysis. The vertical dashed line represents the pooled point estimate of the mean difference. The solid black line indicates the null hypothesis (MD = 0)

Pooled data obtained from 8 RCTs [17, 20–23, 25–27] did not show any significant effect of IER on triglyceride concentrations (WMD: − 3.11 mg/dL, 95% CI − 9.76 to 3.54; p = 0.36) (Fig. 4a). However, subgroup analyses showed a slightly significant triglyceride reduction in the IER arms employing other fasting regimens (WMD = − 14.4 mg/dL 95% CI − 28.6 to − 0.23; p = 0.046; I^2^ = 0%). Characteristics of the “feed” days were not associated with differences in triglyceride changes (Additional file 8). HDL-cholesterol levels increased after IER regimens, albeit not significantly (WMD = 1.72 mg/dL 95% CI − 0.20 to 3.63; p = 0.07) (Fig. 4c). Subgroup analysis revealed a significant HDL-cholesterol increase with a balanced diet on “feed” days (WMD = 2.88 mg/dL 95% CI 0.66 to 5.09; p = 0.011; I^2^ = 0%) compared with ad libitum eating (Additional file 9). No between-arm differences were found for total cholesterol and LDL-cholesterol (Fig. 4b, d). Finally, changes in both SBP and DBP did not significantly differ between arms (Additional file 10).

### Publication bias

We used the Egger’s test for funnel plot asymmetry to detect a potential publication bias on reporting results on weight change. Test result (p = 0.15) did not suggest any asymmetry in the funnel plot (Additional file 11).

### Safety

No major adverse events were reported. Only 1 patient from the IER arm of the RCT supplying 0 kcal during “fast” days developed gallbladder dyskinesia and underwent cholecystectomy after completing the study, but this event was reported to be unrelated to the intervention [27]. Minor physical or psychological adverse effects, such as lack of energy, headaches, feeling cold, constipation, bad breath, lack of concentration, bad temper, were reported in a minority of participants from the IER arms (< 20%) in a few studies [20, 21, 23, 26]. On the other hand, hunger was reported in the first weeks by about half of participants to a 5:2 regimen in 1 trial, but this symptom improved over time [23].

## Discussion

An intermittent regimen of energy restriction (at least 1 day/week) determined a loss in body weight and percent body weight similar to continuous (non-intermittent) energy restriction. Interestingly, a slight reduction in fasting insulin concentrations was evident with IER regimens employing 2 days/week “fast”, but the clinical relevance of this result is uncertain.

### Effects of IER on weight loss and fat mass

Most systematic reviews and meta-analyses demonstrated that IER regimens achieved comparable weight loss as CER regimens [4, 5, 9], reporting an overall weight loss ranging from 4 to 8% [2, 3, 7, 9], and a difference of − 4.14 kg to + 0.08 kg versus the comparator arms [4, 5, 29]. Our results are in accordance, even if the trials previously included differed from ours, since we have included only RCTs with a at least 1 day/week and no more than 6 day/week of “fasting”, and with an extremely low energy supply during the “fast” days. This latter choice derived from the idea of studying conditions simulating as much as possible a condition of fasting, whose benefits, proven by animal studies, seem to depend on the shift in metabolism from glucose utilization and fat synthesis/storage towards reduced insulin secretion and fat mobilization/oxidation [30, 31].

There is no clear definition of IER, and intermittent regimens providing up to 800 kcal [5, 9], with ≥ 7 “fast” days [4, 6, 9, 29], including time-restricted feeding [2, 6–8, 32], with unlimited energy restriction as a comparator group [2, 3, 5–7], or not randomized controlled trials [2] have been included within previous reviews. We have taken care to define precise inclusion criteria to limit variability and increase the comparability among trials, and we have obtained a low heterogeneity.

It could be hypothesized that the very low caloric intake on “fast” days determined an overall lower caloric intake in the IER arms, which were therefore difficult to be compared with the CER arms. In the only RCT where water and calorie-free beverages were allowed in the “fast” days, a significant between-arms difference in energy intake was evident [27]; in two studies a between-arms difference of 300–400 kcal was observed [22, 23] while most RCTs reported a negligible between-arms difference (~ 100 kcal) [17, 20, 21, 25, 26]. Consistently, our sensitivity and subgroup analyses did not find significant between-arms differences.

Furthermore, the percent weight loss was highly overlapping, and no apparent superiority of a dietary regimen was evident. Indeed, participants of the IER arms from all RCTs lost ≥ 5% of their initial weights, thus confirming the clinical usefulness of this approach at least in the short term, i.e. within 24 weeks.

Previous reviews reported a FM loss ranging from 4 to 7% [3] to 11–16% [2] in the IER arms, and the only meta-analysis evaluating this outcome reported a differential loss of 1.38 kg with respect to comparator arms [5]. We failed to find significant between-arms difference for this outcome, suggesting that such a regimen could be a valid, but not superior alternative to CER.

Intriguingly, participants to the IER regimens usually did not consume as much food in the “feed” days as to compensate for the caloric restriction of the “fast” days, thus suggesting that IER could reduce food intake even in the “feed” days, without compensatory overeating [6, 31]. This finding was not confirmed by all studies [28, 33, 34]. Furthermore, adverse events were sometimes higher with the IER regimens [20, 21, 26], and the participants reported stronger feelings of hunger [21, 23]. The compliance and adherence to the intervention diets was heterogeneous among trials, the attrition rate was often higher in the IER arms [17, 22, 24, 26, 31, 35], and the percentage of participants planning to continue with the dietary regimen beyond 6 months was lower in the IER arms [26]. Overall, these data do not support the fact that IER is easier and more acceptable than CER to everyone. Moreover, the reduction in resting energy expenditure, i.e. the compensatory metabolic response which reduces the degree of weight loss, has been reported to be either reduced (favoring weight loss) [27, 36] or increased (attenuating weight loss) [22, 28] with IER regimens. Indeed, some studies suggest that IER evokes the same adaptive response as CER [6, 37].

The hypothesized benefits of IER, extensively studied in animal models, included the use of fats during severe energy restriction with preferential reduction of adipose mass, the stimulation of browning in white adipose tissue, increased insulin sensitivity, lowering of leptin and increased human growth hormone, ghrelin and adiponectin circulating levels, reduced inflammation and oxidative stress [30]. The trigger of adaptive cell response leading to enhanced ability to cope with stress, improved autophagy by sirtuin-1 activity stimulation, modification of apoptosis, increase of vascular endothelial growth factor expression in white adipose tissue, the action on the metabolism via Forkhead Box A genes, and reduction of advance glycation end-products might be all possible metabolic pathways explaining the beneficial effects of IER [7, 30, 38, 39]. In mice, IER determined metabolic improvements and weight loss as a consequence of a shift in the gut microbiota composition, leading to an increase in the production of acetate and lactate and to the selective upregulation of monocarboxylate transporter in beige adipose cells which stimulate beige fat thermogenesis [40]. At present, many of these adaptive mechanisms have been demonstrated in animal experimental models but not in humans, thus more research is still needed.

### Effects of IER on cardiometabolic markers

IER regimens were associated with lower circulating insulin values; a significant reduction was evident for the 5:2 “fasting” regimen only. Indeed, two RCTs, both employing this regimen, determined the difference [20, 26]. Our data are in line with the results of a previous meta-analysis reporting a significantly higher reduction in fasting insulin (− 0.67 µU/mL) in the IER arms [5]. The difference we found (− 0.89 µU/mL) was statistically significant, but not clinically relevant, above all considering the fact that participants to the included RCTs were overweight/obese and therefore probably insulin-resistant individuals.

Our data synthesis on glucose, HOMA-IR, HbA1c showed no between-arms difference. We did not include patients with T2DM from 2 RCTS in the pooled analysis on fasting glucose, since most participants were on hypoglycemic drugs and their glycemic values would be certainly influenced by the treatment [24, 25]. Highly contrasting human studies are available about the benefits of IER on glucose metabolism and insulin sensitivity [3, 6, 31], contrarily to animal studies strongly suggesting a benefit in T2DM prevention [1, 31]. The improvements in glucose homeostasis might be therefore comparable to those obtained by continuous energy restrictions.

Our meta-analysis did not show significant between-arms difference in lipid values and arterial blood pressure, with the exception of a small difference in subgroup analyses on triglyceride concentrations (− 14 mg/dL) and HDL-cholesterol (+ 2.88 mg/dL), not meaningful from a clinical point of view. Most studies showed reduction in triglyceride levels between 15 and 42% in the IER arms [31, 41], and the only available meta-analysis reported a between-arms not significant difference of 2.65 mg/dL [5]. Reduction in total cholesterol, LDL-cholesterol in the IER arms ranged respectively between 6–25%, 7–32%, with small effects on HDL-cholesterol [1, 31], and between-arms differences resulted not significant [5]. Intriguingly, a few studies reported that IER regimens determined an increase in LDL particle size [19, 42], and reduced post-prandial hypertriglyceridemia [22], thus potentially conferring cardio-protection, since the lower the LDL size, the higher the oxidizability and the susceptibility to arterial penetration, and higher post-prandial hyperlipemia is a marker of atherosclerosis progression. Furthermore, fasting can act on many enzymes implicated in lipid and lipoprotein metabolism [27]. However, all these reports need confirmation in larger human RCTs.

Similarly, data on arterial BP were controversial, with the majority of human studies reporting no differences between IER and CER regimens [1, 5, 31, 41]. Indeed, most of the published studies and RCTs included normotensive subjects at baseline, making it difficult to identify differences between-arms.

Therefore, unlike the very promising data on animals, evidence is not sufficiently robust to suggest the superiority of intermittent vs. continuous caloric restriction regimens on the main cardiovascular factors in humans.

### Clinical implications

Weight loss maintenance should be an integral component of the management of obesity, owing to the weight regain usually occurring with time. The 2 RCTs including longer follow-ups (24 months) did not find between-arms differences in weight loss maintenance [17, 27]. Studies with longer follow-ups, evaluating the long-term sustainability, adherence to, and safety of IER regimens are needed. Furthermore, no RCT evaluated hard endpoints, such as cardiovascular outcomes or T2DM incidence. Two observational cohort studies found that fasting was associated with a lower prevalence of coronary artery diseases or diabetes diagnosis but are limited by a lack of a comprehensive dietary history and many potential bias [43, 44]. It could be hypothesized that IER regimens should be proposed in clinical practice, since it is possible that some individuals find easier to reduce their energy intakes for 1 or more days per week, rather than every day. It is well known that a single diet fit not all, and in the choice of the individual’s tailored regimen, IER strategies should be considered by health care professionals. In this way, data on the feasibility of these regimens in “real life” would be obtained.

### Strengths and limitations

This is, to our knowledge, the largest and updated meta-analysis on the effects of IER on weight loss and multiple metabolic outcomes, setting strict inclusion criteria to increase comparability among studies.

The high variability among the RCTs in the feeding protocols, the limited follow-up, the small sample sizes, the high drop-out rates potentially leading to selection bias, the limited reporting of adverse events and blinding of investigators about arm allocation, or other methodological problems are all limitations to be considered. Finally, most studies were performed by the same authors and the majority of subjects included were adult healthy women, thus limiting the generalizability of the results.

## Conclusion

In overweight/obese adults, IER is as effective as CER for promoting weight loss and metabolic improvements in the short term. Further long-term investigations are needed to draw definitive conclusions.

## Additional files

**Additional file 1.** Electronic search strategy.

**Additional file 2.** Risk of bias assessment in the trials included in the systematic review.

**Additional file 3.** Changes in outcomes at the end of the trials.

**Additional file 4.** Subgroup analysis of weight loss based on the type of regimen (a) and on dietary characteristics of the “feed” days (b).

**Additional file 5.** Percent weight loss (a) and subgroup analysis of percent weight loss based on the type of regimen (b) and dietary characteristics of the “feed” days (c).

**Additional file 6.** Meta-analysis of the effects of intermittent energy restriction versus continuous energy restriction on body composition (a) and waist circumference (b).

**Additional file 7.** Subgroup analysis of fasting insulin based on the type of regimen.

**Additional file 8.** Subgroup analysis of triglycerides based on the type of regimen (a) and dietary characteristics of the “feed” days (b).

**Additional file 9.** Subgroup analysis of HDL-cholesterol based on the type of regimen (a) and dietary characteristics of the “feed” days (b).

**Additional file 10.** Meta-analysis of the effects of intermittent energy restriction versus continuous energy restriction on systolic blood pressure (SBP) (a) and diastolic blood pressure (DBP) (b).

**Additional file 11.** Funnel plot for publication bias detection on weight loss changes.

## Abbreviations

BIA: body impedance analysis
BMI: body mass index
BP: blood pressure
CER: continuous energy restriction
CI: confidence interval
CINAHL: Cumulative Index to Nursing and Allied Health Literature
DBP: diastolic blood pressure
DXA: dual X-ray absorptiometry
FFM: fat free mass
FM: fat mass
HbA1c: glycated hemoglobin A1c
HOMA-IR: Homeostasis Model Assessment-Insulin Resistance
IER: intermittent energy restriction
MeSH: medical subject headings
RCTs: randomized controlled trials
Si: insulin-sensitivity index
SBP: systolic blood pressure
T2DM: type 2 diabetes mellitus
WMD: weight mean difference
