# Supplementary material for: Intermittent versus continuous energy restriction on weight loss and cardiometabolic outcomes: a systematic review and meta-analysis of randomized controlled trials
Source: J Transl Med. 2018 Dec 24;16:371. doi: 10.1186/s12967-018-1748-4 (PMC6304782; doi:10.1186/s12967-018-1748-4)
Supplement: Supplementary file 1 — Additional file 1. Electronic search strategy. [file 12967_2018_1748_MOESM1_ESM.docx]

Electronic search strategy

| **PubMed** |
| --- |
| *Filters activated: humans* |
| #1 Intermittent fasting |
| #2 Alternate day fasting |
| #3 Intermittent energy restriction |
| #4 Periodic fasting |
| #5 Intermittent fasting or Alternate day fasting or Intermittent energy restriction or Periodic fasting |
| #6 #5 and Weight loss [MESH] |
| #7 #5 and Weight gain [MESH] |
| #8 #5 and Fat mass |
| # 9 #5 and Obesity [MESH] |
| #10 #5 and Blood Glucose [Mesh] |
| #11 #5 and Insulin [MESH] |
| #12 #5 and “Insulin resistance” [MESH] |
| #13 #5 and “Glycated hemoglobin A” [MESH] |
| #14 #5 and “Insulin sensitivity” [MESH] |
| #15 #5 and “Diabetes Mellitus, Type 2" [MESH] |
| #16 Weight loss [MESH] or Weight gain [MESH] or Obesity [MESH] or Blood Glucose [Mesh] or Insulin [MESH] or “Insulin resistance” [MESH] or “Glycated hemoglobin A” [MESH] or "Diabetes Mellitus, Type 2" [Mesh] or Weight [MESH] |
| #17 #5 and #16 |
| #18 #5 and Cholesterol [MESH] |
| #19 #5 and Triglycerides [MESH] |
| #20 #5 and “Blood pressure” [MESH] |
|  |
| **Cochrane Library** |
| *Filters activated: humans* |
| #1 Intermittent fasting |
| #2 Alternate day fasting |
| #3 Intermittent energy restriction |
| #4 Periodic fasting |
| #5 Intermittent fasting or Alternate day fasting or Intermittent energy restriction or Periodic fasting |
| #6 #5 and Weight loss |
| #7 #5 and Weight gain |
| #8 #5 and Weight |
| #9 #5 and Fat mass |
| #10 #5 and Obesity |
| #11 #5 and Blood glucose |
| #12 #5 and Insulin |
| #13 #5 and Insulin resistance |
| #14 #5 and Glycated hemoglobin |
| #15 #5 AND Insulin sensitivity |
| #16 #5 and Diabetes Mellitus Type 2 |
| #17 #5 and Cholesterol |
| #18 #5 and Triglycerides |
| #19 #5 and Blood pressure |
|  |
| **TRIP database** |
| *Population: humans* |
| #1 Intermittent fasting |
| #2 Alternate day fasting |
| #3 Intermittent energy restriction |
| #4 Periodic fasting |
| #5 Intermittent fasting or Alternate day fasting or Intermittent energy restriction or Periodic fasting |
| #6 #5 and Weight loss |
| #7 #5 and Weight gain |
| #8 #5 and Body weight |
| #9 #5 and Fat mass |
| #10 #5 and Obesity |
| #11 #5 and Blood glucose |
| #12 #5 and Insulin |
| #13 #5 and Insulin resistance |
| #14 #5 and Glycated hemoglobin A |
| #15 #5 AND Insulin sensitivity |
| #16 #5 and Diabetes Mellitus Type 2 |
| #17 #5 and Cholesterol |
| #18 #5 and Triglycerides |
| #19 #5 and Blood pressure |

| **EMBASE** |
| --- |
| *Population: humans* |
| #1 Intermittent fasting |
| #2 Alternate day fasting |
| #3 Intermittent energy restriction |
| #4 Periodic fasting |
| #5 Intermittent fasting or Alternate day fasting or Intermittent energy restriction or Periodic fasting |
| #6 (#1 OR #2 OR #3 OR #4 OR #5) and Weight loss |
| #7 (#1 OR #2 OR #3 OR #4 OR #5) and Weight gain |
| #8 (#1 OR #2 OR #3 OR #4 OR #5) and Body weight |
| #9 (#1 OR #2 OR #3 OR #4 OR #5) and Fat mass |
| #10 (#1 OR #2 OR #3 OR #4 OR #5) and Obesity |
| #11 (#1 OR #2 OR #3 OR #4 OR #5) and Blood glucose |
| #12 (#1 OR #2 OR #3 OR #4 OR #5) and Insulin |
| #13 (#1 OR #2 OR #3 OR #4 OR #5) and Insulin resistance |
| #14 (#1 OR #2 OR #3 OR #4 OR #5) and Glycated hemoglobin A |
| #15 (#1 OR #2 OR #3 OR #4 OR #5) AND Insulin sensitivity |
| #16 (#1 OR #2 OR #3 OR #4 OR #5) and Diabetes Mellitus Type 2 |
| #17 (#1 OR #2 OR #3 OR #4 OR #5) and Cholesterol |
| #18 (#1 OR #2 OR #3 OR #4 OR #5) and Triglycerides |
| #19 (#1 OR #2 OR #3 OR #4 OR #5) and Blood pressure |

| **CINALH** |
| --- |
| *Population: humans* |
| #1 Intermittent fasting |
| #2 Alternate day fasting |
| #3 Intermittent energy restriction |
| #4 Periodic fasting |
| #5 Intermittent fasting or Alternate day fasting or Intermittent energy restriction or Periodic fasting |
| #6 #5 and Weight loss |
| #7 #5 and Weight gain |
| #8 #5 and Body weight |
| #9 #5 and Fat mass |
| #10 #5 and Obesity |
| #11 #5 and Blood glucose |
| #12 #5 and Insulin |
| #13 #5 and Insulin resistance |
| #14 #5 and Glycated hemoglobin A |
| #15 #5 AND Insulin sensitivity |
| #16 #5 and Diabetes Mellitus Type 2 |
| #17 #5 and Cholesterol |
| #18 #5 and Triglycerides |
| #19 #5 and Blood pressure |
