# Supplementary material for: Intermittent versus continuous energy restriction on weight loss and cardiometabolic outcomes: a systematic review and meta-analysis of randomized controlled trials
Source: J Transl Med. 2018 Dec 24;16:371. doi: 10.1186/s12967-018-1748-4 (PMC6304782; doi:10.1186/s12967-018-1748-4)
Supplement: Supplementary file 2 — Additional file 2. Risk of bias assessment in the trials included in the systematic review. [file 12967_2018_1748_MOESM2_ESM.docx]

**Additional file 2. Risk of bias assessment in the trials included in the systematic review**

| **Study** | **Random sequence**  **generation** | **Allocation concealment** | **Blinding** | **Incomplete outcome data** | **Selective reporting** | **Free of other bias** |
| --- | --- | --- | --- | --- | --- | --- |
| Antoni R (2018) | L | L | U | L | H | L |
| Carter S (2016) | L | L | H | L | L | L |
| Catenacci VA (2016) | U | U | U | L | L | L |
| Conley M (2018) | L | L | L | L | L | L |
| Coutinho SR (2017) | L | L | U | L | L | L |
| Harvie MN (2011) | U | U | U | L | L | L |
| Harvie MN (2013) | L | L | L | L | L | L |
| Sundfor TM (2018) | L | L | H | L | L | L |
| Trepanowski JF (2017) | L | L | U | L | L | L |
| Varady KA (2011) | U | U | U | L | H | L |
| Williams KV (1998) | U | U | U | L | L | L |

Criteria defined for quality assessment are based on the Cochrane guidelines. Abbreviations: H, high risk of bias; L, low risk of bias; U, unclear or unrevealed risk of bias.
