# Supplementary material for: Intermittent versus continuous energy restriction on weight loss and cardiometabolic outcomes: a systematic review and meta-analysis of randomized controlled trials
Source: J Transl Med. 2018 Dec 24;16:371. doi: 10.1186/s12967-018-1748-4 (PMC6304782; doi:10.1186/s12967-018-1748-4)
Supplement: Supplementary file 3 — Additional file 3. Changes in outcomes at the end of the trials. [file 12967_2018_1748_MOESM3_ESM.docx]

**Additional file 3. Changes in outcomes at the end of the trials**

| **Author**  **(year)**  **[ref]** | **Body weight**  kg  % | **Waist-c**  Cm | **FM**  **FFM**  Kg | **SBP**  **DBP**  mmHg | **Fasting glucose** mg/dL | **HbA1c**  **%** | **Fasting**  **insulin**  µU/mL | **HOMA-IR**  mmol/L*µU/mL  **Si** 10^4^lU/mLmin | **Total chol** mg/Dl | **HDL-chol**  mg/dL | **LDL-chol**  mg/dL | **TG**  mg/dL |
| --- | --- | --- | --- | --- | --- | --- | --- | --- | --- | --- | --- | --- |
| Antoni R  (2018)  [22] | IER= -4.7  CER= -4.4  IER= -5.3  C= -5.0 | IER= -4  CER= -5 | IER= -3.7  CER=-3.8  IER= -1.0  CER=-0.7 | IER= -12  CER= -2  IER= -5  CER= -5 | IER= +3.6  CER=0 | ND | IER= -1.0  CER= -3.5 | IER= -0.1  CER= -0.1  ND | IER= -7.6  CER= -7.6 | IER= 0  CER=0 | IER= -7.0  CER= -4.0 | IER= -8.5  CER=-9.3 |
| Carter S. (2016)  [24] | IER= -6.0  CER= -4.0  IER= -6.2  CER= -5.6 | ND | IER= -3.8  CER=-4.0  IER=-2.2**§**  CER=-1.1 | ND  ND | ND | IER= -0.6  CER=-0.8 | ND | ND  ND | ND | ND | ND | ND |
| Catenacci AV.  (2016)  [27] | IER= -8.2  CER= -7.1  IER= -8.8**§**  CER= -6.2 | ND | IER=- 3.7  CER=-3.7  IER= -3.2  CER=-2.6 | ND  ND | IER= -6.0  CER= +3.3 | ND | IER= +3.0  CER= -0.2 | ND  IER= +0.1  CER= 0 | IER= -31.8  CER= -21.7 | IER= -4.2  CER=-4.2 | IER= -22.6  CER=-16.9 | IER= -25.0  CER= -2.8 |
| Conley M  (2018)  [23] | IER= -5.3  CER= -5.5  IER= -5.5  CER= -5.4 | IER= -8  CER=-7 | ND | IER= -13.5  CER= -10.2    IER= -0.2  CER= -3.7 | IER= -1.8  CER= -3.6 | ND | ND | ND | IER= -0.4  CER= +7 | IER= +1.3  CER= +0.3 | IER= -3.3  CER= -16.9 | IER=-26.4  CER=  -17.3 |
| Coutinho SR (2017)  [28] | IER= -13.9  CER= -11.8  IER= -12.9  CER= -12.1 | ND | IER=-11.3  CER=-9.6  IER= -2.8  CER=-1.9 | ND  ND | ND | ND | ND | ND  ND | ND | ND | ND | ND |
| Harvie MN (2011)  [26] | IER= -5.7  CER= -4.5  IER= -6.9*  CER= -5.3* | IER= -6  CER= -4 | IER= -4.5  CER=-3.6    IER= -1.2 CER=-0.8 | IER= -3.7 CER= -7.5  IER= -4.3 CER= -5.7 | IER= -1.9  CER= -1.9 | ND | IER= -2.1**§**  CER= -1.1 | IER= -0.4**§**  CER= -0.3  ND | IER= -88.0 CER=-19.0 | IER=0  CER= -3.8 | IER= -11.0 CER= -11.0 | IER=-17.7 CER=-26.5 |
| Harvie MN (2013)  [20] | IER= -5.0 CER= -3.7  IER= -6.3*  CER= -4.3* | IER= -5  CER= -3 | IER=-3.7**§**  CER=-2.0  IER= -1.8  CER=-1.4 | IER= -4.2 CER= -10  ND | IER=-1.9  CER= 0 | IER= -2.2  CER=-2.0 | IER=-1.2 **§**  CER= 0 | IER= -0.4 **§**  CER= 0  ND | IER= -9.5  CER= -19.0 | IER= +2.8  CER= +1 | IER= -5.4 CER= -3.8 | IER=-12.4 CER= -7.2 |
| Sundfor TM  (2018)  [21] | IER= -9.1  CER= -9.4  IER= -8.4*  CER= -8.7* | IER = -3  CER= -3 | ND | IER= -4.9  CER= -5.8    IER= -5.8  CER= -4.7 | IER= +3.6  CER=-3.6 | IER= +0.3  CER=+0.2 | ND | ND  ND | IER= -6.2  CER= +2.7 | IER= +1.9  CER= +2.3 | IER= -6.2  CER=-16.9 | IER=-30.9  CER=  -31.1 |
| Trepanowski JF  (2017)  [17] | Δ=0  IER= -6.8  CER= -6.8 | ND | Δ = +0.9  Δ= +0.6 | Δ = +0.8    Δ = - 0.3 | Δ =-1.4 | ND | Δ = - 0.4 | Δ = +0.07  ND | Δ = +3.4 | Δ = +6.2 | Δ = +2.5 | Δ = -10.5 |
| Varady KA  (2011)  [19] | ND  IER= -5.2  CER= -5.0 | ND | ND  ND | ND  ND | ND | ND | ND | ND  ND | ND | ND | IER= -10.0^  CER= -8.0^ | IER=-17^ |
| Williams KV  (1998)  [25] | IER= -9.6  CER= -5.4  IER= -9.2*  CER= -5.4* | ND | ND  ND | ND  ND | ND | IER= -0.7  CER=-0.2 | IER= -5.3  CER= -6.3 | ND  ND | IER= -11.2  CER=-16.7 | IER= +1.9  CER= -5.8 | IER= -3.4  CER=-6.1 | IER=-101.0  CER=-58.0 |

**§ p<0.05**

* data extrapolated from the text

^ values expressed as percentage (%);

Δ corresponds to between-arms change differences

Abbreviations: Blood Pressure (BP), Body Mass index (BMI), Body Weight (BW), Continuous Energy Restriction (CER), Fat Free Mass (FFM), Fat Mass (FM), Intermittent Energy Restriction (IER), High Density Lipoprotein-cholesterol (HDL-c), Low Density Lipoprotein-cholesterol (LDL-c), No Data (ND), Triglycerides (TG), Type 2 Diabetes Mellitus (T2DM), Waist circumference (Waist-c)
