# Supplementary material for: Intermittent versus continuous energy restriction on weight loss and cardiometabolic outcomes: a systematic review and meta-analysis of randomized controlled trials
Source: J Transl Med. 2018 Dec 24;16:371. doi: 10.1186/s12967-018-1748-4 (PMC6304782; doi:10.1186/s12967-018-1748-4)
Supplement: Supplementary file 10 — Additional file 10. Meta-analysis of the effects of intermittent energy restriction versus continuous energy restriction on systolic blood pressure (SBP) (a) and diastolic blood pressure (DBP) (b). [file 12967_2018_1748_MOESM10_ESM.docx]

**Additional file 10. Meta-analysis of the effects of intermittent energy restriction versus continuous energy restriction on systolic blood pressure (SBP) (a) and diastolic blood pressure (DBP) (b).**

a

b

MD (mean difference) indicate the mean difference on change from baseline of the IER *vs* the CER arms. The plotted points are the mean differences and the horizontal error bars represent the 95% confidence intervals. The grey areas are proportional to the weight of each study in the random-effects meta-analysis. The vertical dashed line represents the pooled point estimate of the mean difference. The solid black line indicates the null hypothesis (MD=0).
