# Supplementary material for: Intermittent versus continuous energy restriction on weight loss and cardiometabolic outcomes: a systematic review and meta-analysis of randomized controlled trials
Source: J Transl Med. 2018 Dec 24;16:371. doi: 10.1186/s12967-018-1748-4 (PMC6304782; doi:10.1186/s12967-018-1748-4)
Supplement: Supplementary file 11 — Additional file 11. Funnel plot for publication bias detection on weight loss changes. [file 12967_2018_1748_MOESM11_ESM.docx]

**Additional file 11. Funnel plot for publication bias detection on weight loss changes.**

The funnel plot shows the observed mean differences (on the x-axis) against standard errors (on the y-axis). In the absence of publication bias, the plotted points form a funnel shape.
